# Carfilzomib in multiple myeloma patients with renal impairment: pharmacokinetics and safety

**DOI:** 10.1038/leu.2013.29

**Published:** 2013-03-01

**Authors:** A Z Badros, R Vij, T Martin, J A Zonder, L Kunkel, Z Wang, S Lee, A F Wong, R Niesvizky

**Affiliations:** 1 M and S Greenebaum Cancer Center, University of Maryland, Baltimore, MD, USA; 2 Washington University School of Medicine, St Louis, MO, USA; 3 The University of California, San Francisco, CA, USA; 4 Karmanos Cancer Institute, Wayne State University, Detroit, MI, USA; 5 Independent Consultant, San Francisco, CA, USA; 6 Onyx Pharmaceuticals, Inc., South San Francisco, CA, USA; 7 Weill Cornell Medical College, New York, NY, USA

**Keywords:** carfilzomib, myeloma, renal impairment, dialysis, proteasome inhibitor, relapsed

## Abstract

This phase 2 study assessed the safety, pharmacokinetics, pharmacodynamics and efficacy of carfilzomib, a selective proteasome inhibitor, in patients with multiple myeloma and varying degrees of renal impairment, including patients on chronic hemodialysis. Patients were grouped by creatinine clearance: >80 ml/min, 50–80 ml/min, 30–49 ml/min, <30 ml/min and chronic hemodialysis. Carfilzomib was administered on days 1, 2, 8, 9, 15 and 16 in 28-day cycles: 15 mg/m^2^ (Cycle 1), 20 mg/m^2^ (Cycle 2) and 27 mg/m^2^ (Cycles 3+). There were no differences in carfilzomib clearance or exposure among patients with normal renal function and any group with renal impairment. Grade 3/4 adverse events (AEs) included anemia (28.0%), thrombocytopenia (20.0%), lymphopenia (18.0%) and fatigue (14.0%). AEs were similar among groups. At 15 mg/m^2^, proteasome inhibition up to 85% was observed and did not differ among groups. Although nearly 50% of patients were refractory to both bortezomib and lenalidomide, end of study partial response or better (overall response rate) was 25.5% with 7.9 months median duration of response. In conclusion, the pharmacokinetics and safety of carfilzomib were not influenced by the degree of baseline renal impairment, including in patients on dialysis, and carfilzomib was well tolerated and demonstrated promising efficacy.

## Introduction

Renal impairment is a frequent and severe complication in patients with multiple myeloma (MM).^1, 2^ The pathology is heterogeneous and includes a variety of factors such as hypercalcemia, cast nephropathy and immunoglobulin light chain damage (usually irreversible) to tubular cells.^3^ The incidence of renal insufficiency in patients with newly diagnosed MM varies by the definition; while 50% of 2380 newly diagnosed patients with MM had impaired renal function as determined by elevated creatinine clearance (CrCl) at diagnosis,^4, 5^ only 15–20% had serum creatinine >2.3 mg/dl. Renal impairment has been associated with poor prognosis and shorter survival in patients with MM, not only because of the advanced state of disease that caused the renal impairment but also because of limited treatment options and dose reductions commonly implemented in these patients that lead to diminished efficacy.^1, 5^ While it is true that renal insufficiency may increase the toxicity of various therapies in MM, recent data suggest that novel agents such as thalidomide and bortezomib are safe and effective in patients with MM and renal failure.^6^

Carfilzomib is a selective proteasome inhibitor that, like bortezomib, primarily inhibits the chymotrypsin-like activity of the proteasome.^7, 8^ In preclinical studies, carfilzomib showed greater selectivity than bortezomib for the proteasome without inhibiting off-target proteases, and had antiproliferative activity in cells resistant to bortezomib.^7^ In previous phase 2 studies, carfilzomib demonstrated durable responses in heavily pretreated patients with relapsed and/or refractory MM including patients with mild to moderate renal impairment.^9, 10^ Unlike results shown with intravenous (IV) bortezomib,^11^ peripheral neuropathy events during treatment with carfilzomib are mild and occur at a low rate.^12^

The current phase 2, open-label, multicenter study was designed to assess the influence of renal impairment on the pharmacokinetics (PK) of carfilzomib in patients with relapsed, refractory and/or progressive MM after at least two prior regimens. Secondary outcomes included safety, tolerability, pharmacodynamic (PDn) measures and efficacy.

## Patients and Methods

### Patient population

Patients with relapsed and/or refractory MM whose disease was progressing after two or more prior lines of therapy were eligible to participate. On the basis of CrCl, roughly estimated using the Cockcroft-Gault equation,^13^ patients were assigned to 1 of 4 groups according to renal function or to a fifth group consisting of patients on dialysis (Table 1).

Additional inclusion criteria included, age ⩾18 years, current measurable disease, life expectancy of >3 months, Eastern Cooperative Oncology Group Performance Status 0–2, adequate hepatic function, total white blood cell count ⩾2000/mm^3^, absolute neutrophil count ⩾1000/mm^3^, hemoglobin ⩾7 g/dl and platelet count ⩾30 000/mm^3^. Patients were required to protect against pregnancy during and for 3 months following the study. Patients were excluded if they had previously received carfilzomib therapy; transfusions or growth factor support within 7 days of the first dose; or radiation therapy or immunotherapy, major surgery or chemotherapy with approved or investigative anticancer therapeutics within 3 weeks of the first dose. The following medical conditions were also exclusion criteria: plasma cell leukemia or other malignancy within the past 3 years, significant neuropathy (Grade 2 with pain, or Grade 3/4), POEMS syndrome, severe congestive heart failure (New York Heart Association Class III–IV), symptomatic ischemic heart disease, uncontrolled hypertension, known or suspected HIV infection, active hepatitis infection, known or suspected cardiac amyloidosis and concomitant myelodysplastic syndrome. Patients for whom oral and/or IV fluid hydration, dexamethasone, or allopurinol was contraindicated were also excluded.

This study is registered with clinicaltrials.gov (NCT00721734). All patients provided written informed consent, and the study protocol was approved by the institutional review boards of participating centers.

### Study design and drug dosing

Carfilzomib was administered by IV infusion over 2–10 min on days 1, 2, 8, 9, 15 and 16 of a 28-day cycle for up to 12 cycles. The dose of carfilzomib in Cycle 1 was 15 mg/m^2^. If this dose was tolerated, it was increased to 20 mg/m^2^ at Cycle 2, and to 27 mg/m^2^ at Cycle 3 and for all subsequent cycles as tolerated. Patients who experienced an adverse event (AE) were permitted a one-level dose reduction of carfilzomib (for example, from 27 mg/m^2^ to 20 mg/m^2^); after 1 cycle at the lower dose and resolution of the AE, the previous dose could be resumed. If toxicity continued or recurred, the patient was either permitted a second dose reduction or was discontinued from the study at the discretion of the treating physician and the study medical monitor. Patients who discontinued treatment were followed for 2 years for survival and disease status unless they withdrew consent. Patients in whom there was continuing clinical benefit after 12 cycles could receive additional treatment with carfilzomib in a separate extension study (PX-171-010, NCT00884312).

On the basis of findings from earlier phase 1 and phase 2 studies,^9, 14^ dexamethasone 4 mg was administered before carfilzomib dosing during Cycle 1 and could be continued in subsequent cycles if treatment-related fever, chills, and/or dyspnea were observed. In addition, patients with less than partial response (PR) after Cycle 2 or less than complete response after Cycle 4 were eligible to receive dexamethasone 20 mg before each dose of carfilzomib to improve response; prophylactic antiviral therapy was also added in these patients. In addition, all patients were required to be well hydrated before dosing with carfilzomib. They were encouraged to consume at least 30 ml/kg/day orally for 48 h before carfilzomib dosing in all cycles; in addition, 250–500 ml of IV fluid were administered before and after each carfilzomib dose during Cycle 1. Hydration could be adjusted in patients on dialysis according to their hydration status. Optional allopurinol was provided to patients considered at risk for tumor lysis syndrome. Patients could receive red blood cell transfusions or supportive care with erythropoietin or darbepoetin in accordance with institutional guidelines.

### PK analyses

Blood for plasma PK analysis was collected on days 1 and 15 of Cycle 1, and on day 15 of Cycle 2, before dosing (*t*=0), at the end of dosing, and at 5, 15 and 30 min and 1, 1.5, 2, 4, 6 and 24 h after dosing; urine samples were collected cumulatively over 0–5 h and 5–24 h on days 1 and 15 of Cycle 1 following administration of carfilzomib to determine carfilzomib concentrations. Carfilzomib concentrations were determined using validated liquid chromatography tandem mass spectrometry (LC MS/MS) methods with a calibration range of 0.300–300 ng/ml for plasma samples and 4.0–2000 ng/ml for urine samples. Plasma concentration of carfilzomib versus time was plotted on days 1 and 15 in Cycle 1, and PK parameters were calculated using a non-compartmental constant infusion method using WinNonlin (Pharsight, Sunnyvale, CA, USA). To determine whether renal impairment affected the PK of carfilzomib, the relationship between renal function status and relevant PK parameters was evaluated. Statistical comparison (analysis of variance) of the *ln*-transformed dose-adjusted PK parameters was also performed between patients with normal renal function and each group of patients with renal impairment. For these parameters, 90% confidence intervals (CI) were calculated for the ratios between least squares of the mean (LSM) for each group versus the normal renal function group (Group 1). Two criteria were to be met to conclude that the available data refuted an effect of renal impairment on the PK of carfilzomib: (1) the 90% CI for the LSM ratio of carfilzomib AUC fell within the interval of 80–125%, and (2) the 90% CI for the LSM ratio of Cmax fell within the interval of 70–143%. The primary PK parameters—apparent plasma clearance, dose-normalized AUCinf (total exposure), and the dose-normalized Cmax on day 1 and day 15 of Cycle 1—were also estimated as a function of CrCl by linear regression using a mixed-effects model (Y=*β*0+*β*1 × CrCl+*β*2 × Age+*β*3 × weight, where Y=PK parameter, *β*0 = intercept, and β1, β2 and β3 are slope parameters associated with CrCl, age and weight, respectively).

### PDns

Proteasome chymotrypsin-like activity was measured in whole-blood (red blood cells, RBC) and isolated peripheral blood mononuclear cell samples using a fluorogenic-based substrate assay as previously described.^15^ Blood for PDn analysis was collected before carfilzomib dosing and 1 h after carfilzomib dosing on days 1, 2 and 8 of Cycle 1 and on day 1 of Cycle 2.

### Safety

All patients who received at least one dose of carfilzomib were included in the safety population. Incidence, severity and duration of AEs, including all serious AEs and those considered to be treatment related, and shifts to or from abnormal relative to baseline in key laboratory parameters were categorized according to the National Cancer Institute's Common Terminology Criteria for Adverse Events (CTCAE) Version 3.0.^16^ In addition, all patients had intensive triplicate electrocardiogram (ECG) readings before carfilzomib dosing, 5 and 20 min post-dose, and 1, 2, 4 and 24 h post-dose on days 1 and 15 of Cycle 1 and on day 15 of Cycle 2 that were read by a central laboratory. ECGs were performed in most patients to satisfy regulatory requirements, but were not required by the study protocol.

### Efficacy

Patients who received at least 1 cycle of carfilzomib and had at least one post-baseline assessment for disease response were included in the efficacy analysis. Each evaluable patient's best response to treatment over the course of the study was evaluated by the investigator and sponsor using the International Myeloma Working Group Uniform Response Criteria, modified to include minimal response defined per the European Group for Blood and Marrow Transplantation.^17, 18^ Responses assessed by the investigators at the end of the study are reported here. The overall response rate was defined as the proportion of PR or better responses, and the 95% Clopper-Pearson exact binomial CI was calculated. The duration of response was calculated as the time from the first evidence of a PR or better until confirmation of disease progression. Median time to progression and progression-free survival were assessed among all patients with >stable disease (s.d.). For progression-free survival, deaths due to causes other than disease progression were censored.

## Results

### Patient characteristics

A total of 50 patients were enrolled in the study at five centers in the US; the first patient in November 2008 and the last patient in January 2010. PK, safety, and PDn results are based on the data collected through March 2010, 2 months after the enrollment completed. End-of-study response data (overall response rate and duration of response) are based on assessments through December 2011. Patient demographics and baseline characteristics are shown in Table 2. Approximately 26.0% of patients had a poor prognosis as assessed by cytogenetic analysis and/or fluorescence *in situ* hybridization. All but two patients (96.0%) had previously received bortezomib, and 33 patients (66.0%) were refractory to prior bortezomib therapy, including 17 patients (34.0%) who were refractory to bortezomib as the most recent therapy. Twenty-three patients (46.0%) were refractory to both bortezomib and lenalidomide.

### Patient disposition

Patients received a median of 4 cycles (range, 1–12) of carfilzomib. All 50 patients were included in the safety analysis, 10 patients were not evaluable for PK, and three were not evaluable for efficacy (Table 1). Thirteen patients continued to receive treatment on study at the time of the data cutoff, and three additional patients completed 12 cycles of carfilzomib treatment and continued to receive carfilzomib on extension access protocol PX-171-010.^19^ Among the 34 patients who discontinued treatment, 24 discontinued due to PD, 6 discontinued due to an AE, and 4 withdrew consent.

The doses of carfilzomib across groups ranged from 14.3 mg/m^2^to 25.3 mg/m^2^ per administration throughout the study and were generally consistent across the groups. Forty-one patients (82.0%) escalated to 20 mg/m^2^ in Cycle 2, and 27 of these (54.0% overall) escalated to 27 mg/m^2^ in Cycle 3. Twenty-two patients (44.0%) missed a carfilzomib dose, and 17 (34.0%) had carfilzomib administration delayed at least once. Exposure to carfilzomib was similar between groups with two exceptions—those on chronic dialysis and those in Group 3 with moderate renal impairment received fewer cycles primarily due to discontinuations attributed to progressive disease. Per protocol, 28 patients with <PR after Cycle 2 or <complete response after Cycle 4 received dexamethasone 20 mg before each dose of carfilzomib to improve response.

### Pharmacokinetics

**Figure 1:**
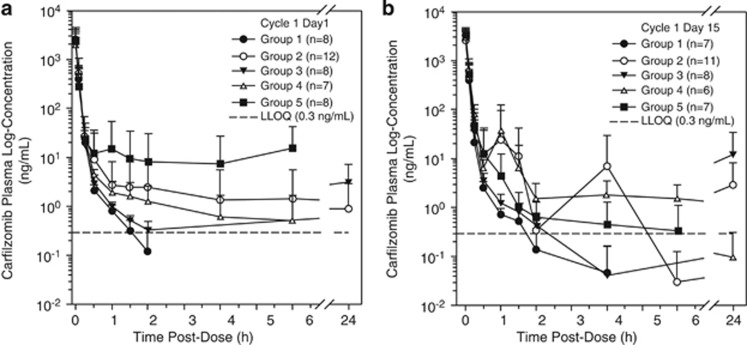
Carfilzomib plasma concentration after administration of 15 mg/m^2^ in Cycle 1. (**a**) Profiles on day 1 by group. (**b**) Profiles on day 15 by group. Group 1, normal renal function; Group 2, mild renal impairment; Group 3, moderate renal impairment; Group 4, severe renal impairment; Group 5, chronic dialysis.

Table 3 shows the PK parameters for the five groups after dosing. Following IV administration of 15 mg/m^2^ over 2–10 min on day 1 of Cycle 1, carfilzomib plasma concentration decreased rapidly in a biphasic manner with a median terminal half life (t1/2) of less than 60 min across all groups, and the profile was consistent between days 1 and 15 of Cycle 1 (Figure 1). Mean (±s.d.) carfilzomib clearance ranged from 113 ±40.7 l/h (Group 2) to 288 ±264 l/h (Group 3). Across groups, the geometric means of Cmax and AUCinf ranged from 1231–2077 ng/ml and 145–241 ng·h/ml, respectively. Analysis of variance of the *ln*-transformed dose-adjusted plasma PK parameters indicated that there were no apparent differences in the Cmax and AUCinf between Group 1 and any of the groups of patients with renal impairment. However, due to high interpatient variability, none of the 90% CIs for the ratio of geometric LSM of AUC were entirely contained within the prespecified interval of 80–125% and none of the 90% CIs for the ratio of geometric LSM of Cmax were entirely contained within the prespecified interval of 70–143%.

As a secondary analysis of the impact of renal impairment on carfilzomib clearance, the carfilzomib PK profile was estimated as a function of CrCl by linear regression using a mixed effects model. Carfilzomib clearance, dose-normalized Cmax, and dose-normalized AUCinf on day 1 of Cycle 1 all had a slope of the regression that did not significantly (*P*>0.05) differ from zero (Table 4). The same was true when adjusted for age, weight, or age+weight. Although the analysis of variance analysis was inconclusive, these results suggest that renal function did not affect carfilzomib PK. A robust comparison between dosing cycles could not be performed due to the limited PK data obtained from patients who were treated with 15 mg/m^2^ for Cycle 2. In treatment Cycle 2, patients were allowed to escalate the dose to 20 mg/m^2^.

A summary of PK results for the carfilzomib 20 mg/m^2^ dose on day 15 of Cycle 2 is also presented in Table 3. Similar to the 15 mg/m^2^ dose, the AUC, Cmax, and t1/2 across the groups of patients at 20 mg/m^2^ suggest no apparent effect of renal impairment on the PK of carfilzomib in patients with MM.

All but two patients had measurable concentrations of carfilzomib in urine during the 0- to 5-h post-dose collection interval on days 1 and 15 of Cycle 1. Carfilzomib concentration was below the lower limit of quantitation for most patients during the 5- to 24-h post-dose interval on all days. On day 1 of Cycle 1, the mean total concentration during this time period ranged from 0.050 mg in the group with moderate renal impairment to 0.157 mg in the group with normal renal function. In all groups, the total amount of carfilzomib recovered in the urine on Days 1 and 15 of Cycle 1 represented less than 1% of the administered dose, suggesting renal clearance does not play an important role in carfilzomib elimination.

### Safety

There were no appreciable differences in the safety profiles of carfilzomib among Groups 1–4 for frequency or National Cancer Institute's Common Terminology Criteria for Adverse Events grade of AEs (Table 5). All but one patient in Group 1 (normal renal function) and two patients in Group 5 (dialysis) experienced at least one treatment-emergent AE (94.0% overall). The most common AEs of any grade were fatigue (56.0%), anemia (50.0%), nausea and diarrhea (36.0% each) (Table 5). Hematologic abnormalities were typically transient, as suggested by the relatively consistent median laboratory values over the course of treatment (Supplementary Figure 1). Clinical congestive heart failure was noted in four patients, three of whom continued on the study after fluid adjustment, and one of whom discontinued.

Serious AEs occurred in 33 patients (66.0%), the most common being pneumonia (*n*=8 patients, 16.0%), acute renal failure, congestive heart failure, dehydration, and influenza (three patients each, 6.0%). Most Grade 3/4 AEs were hematologic, including anemia (28.0%), thrombocytopenia (20.0%) and lymphopenia (18.0%) (Table 5). Grade 3/4 infections included bacterial pneumonia (*n*=6, 12.0%), influenza (*n*=3, 6.0%), respiratory tract infection (*n*=2, 4.0%) and sepsis (*n*=2, 4.0%).

Renal events were assessed based on changes in serum creatinine using National Cancer Institute's Common Terminology Criteria for Adverse Events grading. Five patients (not including those on dialysis) experienced elevations in serum creatinine from Grade ⩽2 at baseline to Grade 3 at any point after beginning treatment. Six patients (12.0%) with pre-existing renal impairment (in Groups 2, 3 and 4) experienced worsening of renal function to Grade 3: one patient in Group 3 had increased blood creatinine, and five patients had acute renal failure. The five renal failure events were preceded by clinical or laboratory evidence of myeloma progression, while the serious AEs of increased blood creatinine followed an episode of infection and possible dehydration.

Neuropathy was noted at baseline in 92.0% of patients: Grade 1 in 33 patients (66.0%) and Grade 2 in 7 patients (26.0%). The incidence of treatment-emergent or worsening neuropathy (a composite of AE terms ‘peripheral neuropathy,' ‘neuropathy', ‘peripheral sensory neuropathy' and ‘peripheral motor neuropathy') of any grade was 12.0%. Only one patient (who had Grade 2 neuropathy at baseline) reported Grade 3 neuropathy during the study (2.0%), which did not resolve before data cutoff; no other Grade 3 neuropathy was reported.

Forty-nine patients had evaluable ECGs performed in triplicate while on study. The ECG data showed no clinically relevant effects on heart rate, atrioventricular conduction, or cardiac depolarization as measured by the QT, PR, and QRS intervals, and no correlation with the plasma concentration of carfilzomib. However, the results could not clearly define the magnitude of effect due to the small sample size and lack of a control group.

AEs led to dose reduction in nine patients (18.0%) two each in Groups 1, 3, and 4, and three patients in Group 2. AEs leading to treatment discontinuation in six patients included (one patient each) congestive heart failure, fatigue, dyspnea, hypoesthesia, sepsis and venoocclusive disease of the liver. The latter followed a severe respiratory infection requiring intubation and intensive care support. All AEs leading to treatment discontinuation were attributed to carfilzomib.

There were five deaths on study or within 30 days of discontinuation of treatment: one patient each in Group 2 and Group 4, and three patients in Group 5. All deaths were attributed primarily to disease progression, with one death complicated by a respiratory tract infection.

### PDns

**Figure 2:**
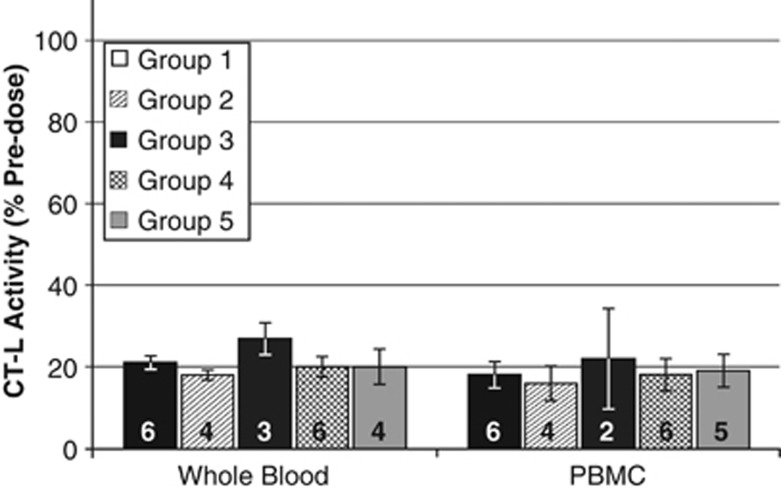
Proteasome inhibition following the first dose of carfilzomib. Proteasome activity was measured in whole-blood and peripheral blood mononuclear cells before and 1 h after the first dose of 15 mg/m^2^ carfilzomib on day 1 of Cycle 1 (using LLVY-AMC substrate). Data are presented as mean activity (±s.e.m.) relative to pre-dose values for each group. The number of patient samples for each Group is noted. Group 1, normal renal function; Group 2, mild renal impairment; Group 3, moderate renal impairment; Group 4, severe renal impairment; Group 5, chronic dialysis.

One hour after the first dose of carfilzomib (15 mg/m^2^), proteasome CT-L activity was significantly inhibited (by 73–87%) from pre-dose levels in RBCs and peripheral blood mononuclear cells. The extent of inhibition did not differ significantly among groups (Figure 2). Proteasome inhibition was maintained in peripheral blood mononuclear cells on day 2, but recovery of activity was complete or near to complete on day 8 (5 days after the most recent dose) and at the beginning of Cycle 2 (12 days after dosing). Cumulative inhibition of proteasome CT-L activity was noted in RBCs during the first treatment cycle with little to no recovery by the start of Cycle 2.

### Efficacy

Twelve of 47 response-evaluable patients achieved PR or better (overall response rate 25.5%) (Table 6). The median duration of response was 7.9 months (95% CI 6.5–not reached). Renal impairment did not appear to diminish antitumor response to carfilzomib; among the 36 response-evaluable patients with renal impairment (excluding Group 1, patients with normal renal function), overall response rate was 27.7%.

Twenty-eight patients received dexamethasone before each carfilzomib dose after Cycle 2 to improve responses (Table 6); 26 patients received dexamethasone 20 mg, while two patients received dexamethasone 40 mg. Response improved to PR after the addition of dexamethasone in nine of these 28 patients (32.1%).

## Discussion

This is the first study that directly assessed carfilzomib PK and PDn in patients with MM and renal impairment, including patients on dialysis. Carfilzomib PK did not appear appreciably altered in patients with renal impairment, including those on dialysis. The rapid systemic clearance and short half-life (t1/2 <60 min) in patients with varying degrees of renal impairment were consistent with those observed in other PK studies of carfilzomib in patients with hematologic or solid tumor malignancies.^14, 20^ Concordant with the observation that renal clearance is not a significant pathway for carfilzomib elimination, carfilzomib recovered in urine samples within 24 h post-dosing represented <1% of the total dose. The results of this study demonstrate that carfilzomib can be safely administered at the same dose to patients with MM regardless of their renal function.

The small number of patients in each group produced a very large 90% CI in each PK comparison, due in large part to the interpatient variability associated with rapid drug clearance. As observed in animal studies,^21^ carfilzomib plasma concentration declined rapidly immediately following the end of dosing. Consequently, small differences in the timing of plasma sampling at the end of dosing can substantially impact the observed Cmax, which will then affect the calculated PK parameters including AUC and clearance. In addition, variable infusion rate (infusion duration from 2 to 10 min) will affect Cmax. Thus the CIs of the LSM ratios for AUC and Cmax did not fall within the prespecified ranges. In support of this analysis, a sensitivity analysis using the mixed-effects linear regression model demonstrated that renal function, as measured by CrCl, was not a statistically significant factor affecting the observed PK parameters.

In the current trial in patients with varying degrees of renal impairment, there was no appreciable difference in the type, frequency, or severity of AEs compared with other carfilzomib phase 2 trials, where patients had CrCl ⩾30 ml/min.^9, 10, 14, 22, 23^ It should be noted that the challenges in administering the drug were manageable. Most cases of congestive heart failure were related to aggressive hydration and three patients safely continued on the study once hydration was adjusted, suggesting, at least clinically, that the drug does not have direct cardiac effects. In addition, ECGs showed no changes related to carfilzomib, regardless of the level of renal impairment. As seen in all phase 2 MM studies with carfilzomib to date, Grade ⩾3 AEs in the present trial were predominantly related to hematologic, respiratory, constitutional and electrolyte systems.^24^ Likewise, the low rate of treatment-emergent neuropathy in this study is consistent with previous studies with single-agent carfilzomib, providing further evidence that carfilzomib does not cause or exacerbate neuropathy.^12^ The case of venoocclusive liver disease is probably a random or multifactorial event that followed respiratory failure requiring intubation complicating an infection and right ventricular failure. No other cases of venoocclusive disease have been observed in over 500 patients treated in phase 2 trials.

Most patients who experienced irreversible worsening of renal function had clear evidence of progressive myeloma. The dose of carfilzomib was safely escalated to 27 mg/m^2^—the dose used in patients with normal renal function—and did not appear to be associated with clinically relevant nephrotoxicity. This is different from what is reported for other therapies for MM such as high-dose melphalan and lenalidomide.^25^ Both are dependent on renal function for clearance, and dose modification is recommended based on severity of renal impairment to minimize hematologic toxicity. The results of the current study suggest that carfilzomib, like bortezomib,^26^ is not likely to require dose or schedule modifications in patients with MM and renal impairment.

The PD analysis confirms prolonged and substantial (73–87%) proteasome inhibition by carfilzomib in patients with renal impairment, as seen in previous studies.^14, 15^ In support of this, among these patients with relapsed and refractory MM and various degrees of renal impairment, treatment with carfilzomib resulted in clinically significant responses. Notably, one of the patients on hemodialysis who had a PR also had improvement in renal function that enabled him to discontinue dialysis. The overall responses noted are quite impressive in these heavily pretreated patients, approximately half of whom were refractory to both bortezomib and lenalidomide. Interestingly, similar to results reported with bortezomib,^27^ improved responses were noted in roughly one-third of patients who received dexamethasone ⩾20 mg (40 mg per week). Moreover, the addition of dexamethasone did not increase overall toxicity of carfilzomib.

In summary, the results of this open-label, nonrandomized study indicate that carfilzomib dose and treatment schedule do not need to be adjusted in patients with renal impairment. The study design and subgroups were maximized to detect differences in PK findings and safety signals, as opposed to efficacy. Further clinical evaluations to substantiate and extend these initial findings are ongoing, including evaluating the impact of renal impairment on combination therapy (for example, carfilzomib with lenalidomide and low-dose dexamethasone, CRd), which has shown promising safety and efficacy to date in patients with MM.^28, 29^

## Figures and Tables

**Table 1:** Group assignment based on renal function and populations for analysis

*Group*	*Renal function status*	*Creatinine clearance, ml/min*^a^	*Study populations*				
			*Enrolled*	*Safety*	*Efficacy*	*PK*	*PDn*
1	Normal	>80	12	12	11	12	6
2	Mild impairment	50–80	12	12	11	9	4
3	Moderate impairment	30–49	10	10	9	7	3^b^
4	Severe impairment	<30	8	8	8	6	6
5	Chronic dialysis		8	8	8	6	5^c^
Total			50	50	47	40	24

Abbreviations: PK, pharmacokinetics; PDn, pharmacodynamics.

a Values between categories were rounded to the nearest integer.

b Samples from two patients were used for PDn analysis in peripheral blood mononuclear cells.

c Samples from four patients were used for PDn analysis in red blood cells.

**Table 2:** Patient characteristics (*n*=50)

*Characteristic*	
Median age, years (range)	64 (45–85)
Male, n (%)	28 (56.0)
Median time since diagnosis, years (range)	6.3 (0.9–19.4)
	
*Immunoglobulin subtype, n* (%)	
IgG	29 (58.0)
IgA	5 (10.0)
IgD	1 (2.0)
Missing or light chain only	15 (30.0)
	
*FISH or cytogenetics, n* (%)	
Normal/favorable	32 (64.0)
Unfavorable (poor prognosis)	13 (26.0)
Unknown/not done	5 (10.0)
	
*Disease Status, n* (%)	
Refractory to last prior regimen	43 (86.0)
Relapsed after last prior regimen	5 (10.0)
No sign of progression at baseline	2 (4.0)
	
*Prior treatment*	
Median number of prior therapies (range)	5 (1–15)
*Prior therapies, n* (%)	
Corticosteroids	50 (100.0)
Bortezomib	48 (96.0)
Lenalidomide	44 (88.0)
Thalidomide	43 (86.0)
Alkylating agents	40 (80.0)
Stem cell transplant	34 (68.0)
Anthracyclines	27 (54.0)

Abbreviations: FISH, fluorescence *in situ* hybridization; Ig, immunoglobulin.

**Table 3:** Summary of PK parameters of carfilzomib in plasma after carfilzomib 15 mg/m^2^ in Cycle 1, or 20 mg/m^2^ in Cycle 2

*Plasma PK*	*Group 1*	*Group 2*	*Group 3*	*Group 4*	*Group 5*
*parameters*^a^					
*Day 1, Cycle 1*	(n=*8*)	(n=*9*)	(n=*5*)	(n=*5*)	(n=*8*)
CL (l/h)^b^	151 (79.3)	113 (40.7)	288 (264)	170 (58.4)	170 (60.2)
AUCinf (h·ng/ml)^b^	233 (51.6)	241 (32.4)	145 (111)	172 (35.6)	193 (55.2)
Cmax (ng/ml)	2077 (91.4)	1623 (161)	1840 (92.4)	1231 (139)	1539 (92.7)
t1/2 (h)^b^	0.398 (0.375–0.626)	0.535 (0.268–2.54)	0.626 (0.544–0.633)	0.890 (0.494–3.77)	0.970 (0.516–4.83)
*Day 15, Cycle 1*	(n=*7*)	(n=*8*)	(n=*5*)	(n=*4*)	(n=*6*)
CL (l/h)^c^	660 (1134)	115 (34.7)	119 (16.5)	110	114 (61.2)
AUCinf (h·ng/ml)^c^	127 (240)	236 (44.3)	257 (10.9)	218	272 (46.4)
Cmax (ng/ml)	1768 (179)	2406 (52.3)	2627 (31.8)	1914 (99.8)	3236 (34.4)
t1/2 (h)^c^	0.481 (0.358–1.73)	0.778 (0.295–0.916)	0.557 (0.531–1.36)	10.7	0.889 (0.357–2.85)
*Day 15, Cycle 2*	(n=*6*)	(n=*7*)	(n=*2*)	(n=*3*)	(n=*4*)
CL (l/h)^d^	123 (28.4)	160 (99.1)	NC	81.7 (47.1)	100 (25.0)
AUCinf (h·ng/ml)^d^	340 (21.3)	246 (52.4)	NC	474 (87.1)	374 (44.4)
Cmax (ng/ml)	4026 (36.2)	2679 (67.0)	2401 (114)	3499 (134)	3384 (29.8)
t1/2 (h)^d^	0.579 (0.284–2.50)	0.568 (0.486–3.02)	NC	6.57 (3.97–9.16)	0.732 (0.570–0.893)

a CL: arithmetic mean (s.d.); AUCinf and Cmax: geometric mean (CV%); t1/2: median (range).

b *n*=6 (Group 1), *n*=6 (Group 2), *n*=3 (Group 3), *n*=4 (Group 4), *n*=5 (Group 5).

c *n*=5 (Group 1), *n*=5 (Group 2), *n*=3 (Group 3), *n*=1 (Group 4), *n*=5 (Group 5).

d *n*=5 (Group 1); *n*=6 (Group 2); *n*=0 (Group 3); *n*=2 (Group 5). NC, value cannot be calculated.

**Table 4:** Linear regression of PK parameters as a function of creatinine clearance

*Parameter*	n	*Slope (95*% *CI)*^a^	P-*value for slope*
Carfilzomib clearance (l/h)	19	−0.607 (−2.129, 0.914)	0.4114
Cmax (ng/ml)	27	0.225 (−0.242, 0.692)	0.3299
AUClast (h·ng/ml)	27	0.008 (−0.031, 0.048)	0.6635
AUCinf	19	0.012 (−0.030, 0.054)	0.5496

Abbreviation: CI, confidence interval.

a Not adjusted for age or weight.

**Table 5:** Incidence and severity of treatment-emergent adverse events of all grades (⩾25%) and Grades 3/4 (⩾5%) (n=50)

	*Group 1* (n=*12*)	*Group 2* (n=*12*)	*Group 3* (n=*10*)	*Group 4* (n=*8*)	*Group 5* (n=*8*)	*Total* (n=*50*) (%)
*All grades*						
** **Hematologic						
** **Anemia	4	7	7	5	2	25 (50.0)
** **Thrombocytopenia	1	6	4	2	2	15 (30.0)
** **Nonhematologic						
** **Fatigue	8	8	6	4	2	28 (56.0)
** **Diarrhea	4	6	5	2	1	18 (36.0)
** **Nausea	5	3	6	1	3	18 (36.0)
** **Hypokalemia	4	3	4	2	3	16 (32.0)
** **Constipation	1	6	5	1	2	15 (30.0)
** **Hypomagnesemia	3	4	4	2	2	15 (30.0)
** **Dyspnea	3	4	4	2	1	14 (28.0)
** **Peripheral edema	4	2	3	3	1	13 (26.0)
*Grades 3/4*						
** **Hematologic						
** **Anemia	2	4	5	2	1	14 (28.0)
** **Thrombocytopenia	1	5	3	1	0	10 (20.0)
** **Lymphopenia	2	3	1	2	1	9 (18.0)
** **Decreased lymphocyte count	1	1	0	0	2	4 (8.0)
** **Decreased platelet count	1	0	2	0	1	4 (8.0)
** **Non-hematologic						
** **Fatigue	2	1	2	1	1	7 (14.0)
** **Pneumonia	1	1	3	1	0	6 (12.0)
** **Pain	0	2	3	0	0	5 (10.0)
** **Increased blood creatinine	0	0	3	1	0	4 (8.0)
** **Dyspnea	0	0	2	1	1	4 (8.0)
** **Decreased hemoglobin	1	0	1	1	1	4 (8.0)
** **Hypokalemia	1	1	0	1	1	4 (8.0)

**Table 6:** Best responses and efficacy end points (*n*=47)

	*Group 1*	*Group 2*	*Group 3*	*Group 4*	*Group 5*	*All patients*
*All patients with response assessment*						
Response category, *n* (%)	*n*=11	*n*=11	*n*=9	*n*=8	*n*=8	*n*=47
Complete response	0	0	0	0	0	0
Very good PR	0	0	0	0	0	0
PR	2 (18.2)	3 (27.3)	2 (22.2)	2 (25.0)	3 (37.5)	12 (25.5)
Minimal response	1 (9.1)	1 (9.1)	0	1 (12.5)	0	3 (6.4)
Stable disease	7 (63.6)	3 (27.3)	4 (44.4)	3 (37.5)	4 (50.0)	21 (44.7)
Progressive disease	1 (9.1)	4 (36.4)	3 (33.3)	2 (25.0)	0	10 (21.3)
Not evaluable	0	0	0	0	1 (12.5)	1
Overall response rate, *n* (%)	2 (18.2)	3 (27.3)	2 (22.2)	2 (25.0)	3 (37.5)	12 (25.5)
Duration of response, median (95% CI), months	NE (2.0–NE)	NE (4.2–NE)	NE (2.3–NE)	NE (7.9–NE)	7.9 (6.5–8.5)	7.9 (6.5–NE)
						
*Response assessment in patients who received dexamethasone ⩾20 mg before carfilzomib doses*^a^						
Response category, *n* (%)	*n*=7	*n*=8	*n*=4	*n*=5	*n*=4	*n*=28
Complete response	0	0	0	0	0	0
Very good PR	0	0	0	0	0	0
PR	3 (42.9)	3 (37.5)	1 (25.0)	0	3 (75.0)	10 (35.7)
Minimal response	1	1 (12.5)	0	1 (20.0)	0	3 (10.7)
Stable disease	3 (42.9)	1 (12.5)	3 (75.0)	2 (40.0)	1 (25.0)	10 (35.7)
Progressive disease	0	3 (37.5)	0	2 (40.0)	0	5 (17.9)
Not evaluable	0	0	0	0	0	0

Abbreviations: CI, confidence interval; NE, not estimated due to censoring; PR, partial response.

a Dexamethasone 20 mg, administered before each carfilzomib dose, was added to treatment at the investigator's discretion to improve response.

## Notes

Ravi Vij: Consultancy and research funding for Onyx Pharmaceuticals. Thomas Martin: Honoraria for Celgene; Consultancy for Onyx Pharmaceuticals. Jeffrey Zonder: Consultancy for Millennium and Amgen; Speakers Bureau for Millennium and Celgene; Research Funding for Millennium; Honoraria for Medtronics. Lori Kunkel: Consultancy for VLST Biotech, Threshold, and Onyx Pharmaceuticals. Zhengping Wang: Employed by and Equity Ownership in Onyx Pharmaceuticals. Susan Lee: Employed by and Equity Ownership in Onyx Pharmaceuticals. Alvin Wong: Employed by and Equity Ownership in Onyx Pharmaceuticals. Ruben Niesvizky: Consultancy and research funding for Celgene, Millennium, and Onyx. Speakers Bureau for Celgene and Millennium. The remaining authors declare no conflict of interest.

## Supplementary Material

Supplementary Figure 1A

Supplementary Figure 1B

Supplementary Figure 1C

Supplementary Figure 1D
