# Supplementary figures and images for: Carfilzomib in multiple myeloma patients with renal impairment: pharmacokinetics and safety
Source: Leukemia. 2013 Mar 1;27(8):1707–14. doi: 10.1038/leu.2013.29 (PMC3740399; doi:10.1038/leu.2013.29)

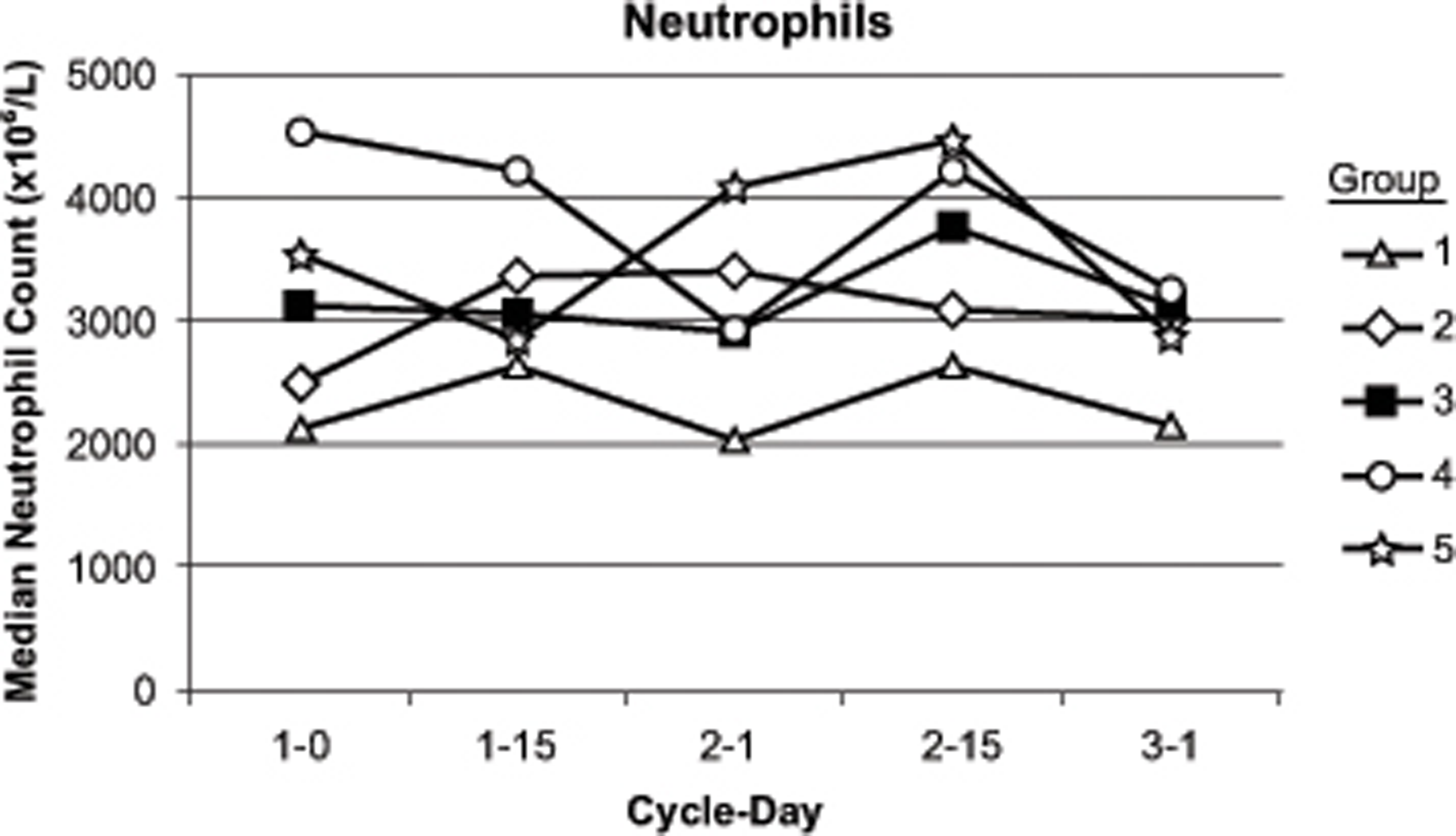

Supplement: Supplementary Figure 1A [file leu201329x1.tif]

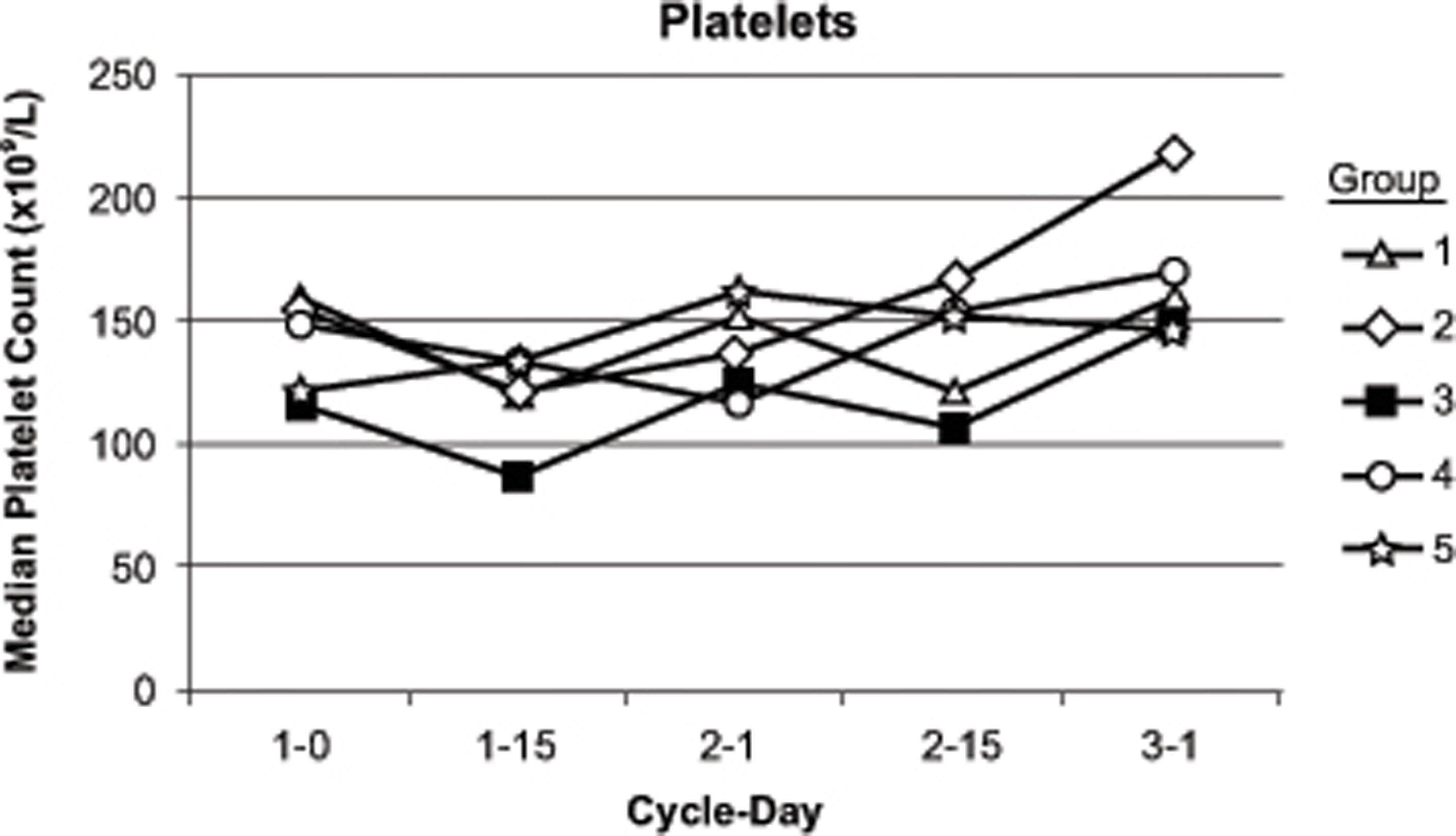

Supplement: Supplementary Figure 1B [file leu201329x2.tif]

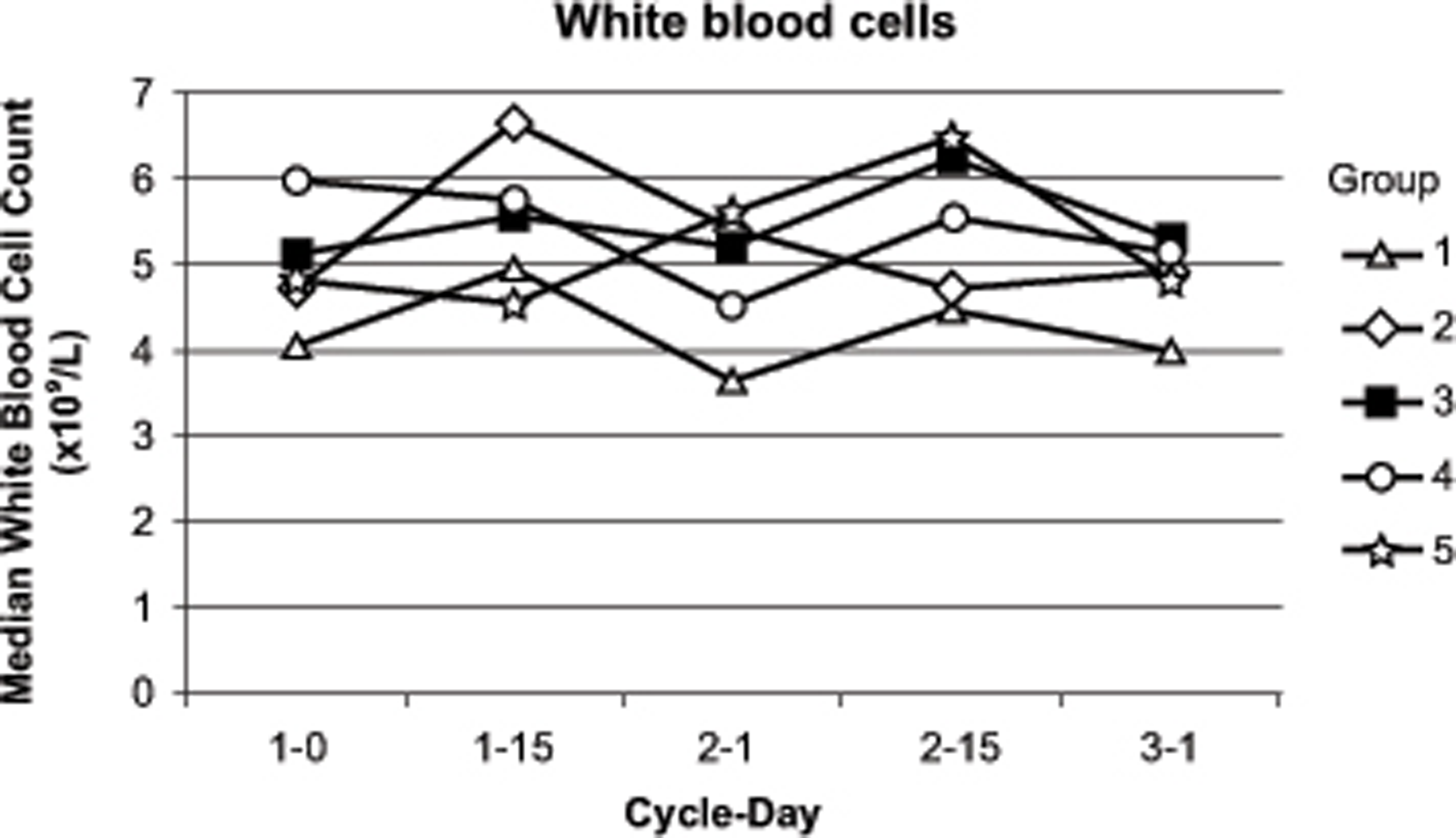

Supplement: Supplementary Figure 1C [file leu201329x3.tif]

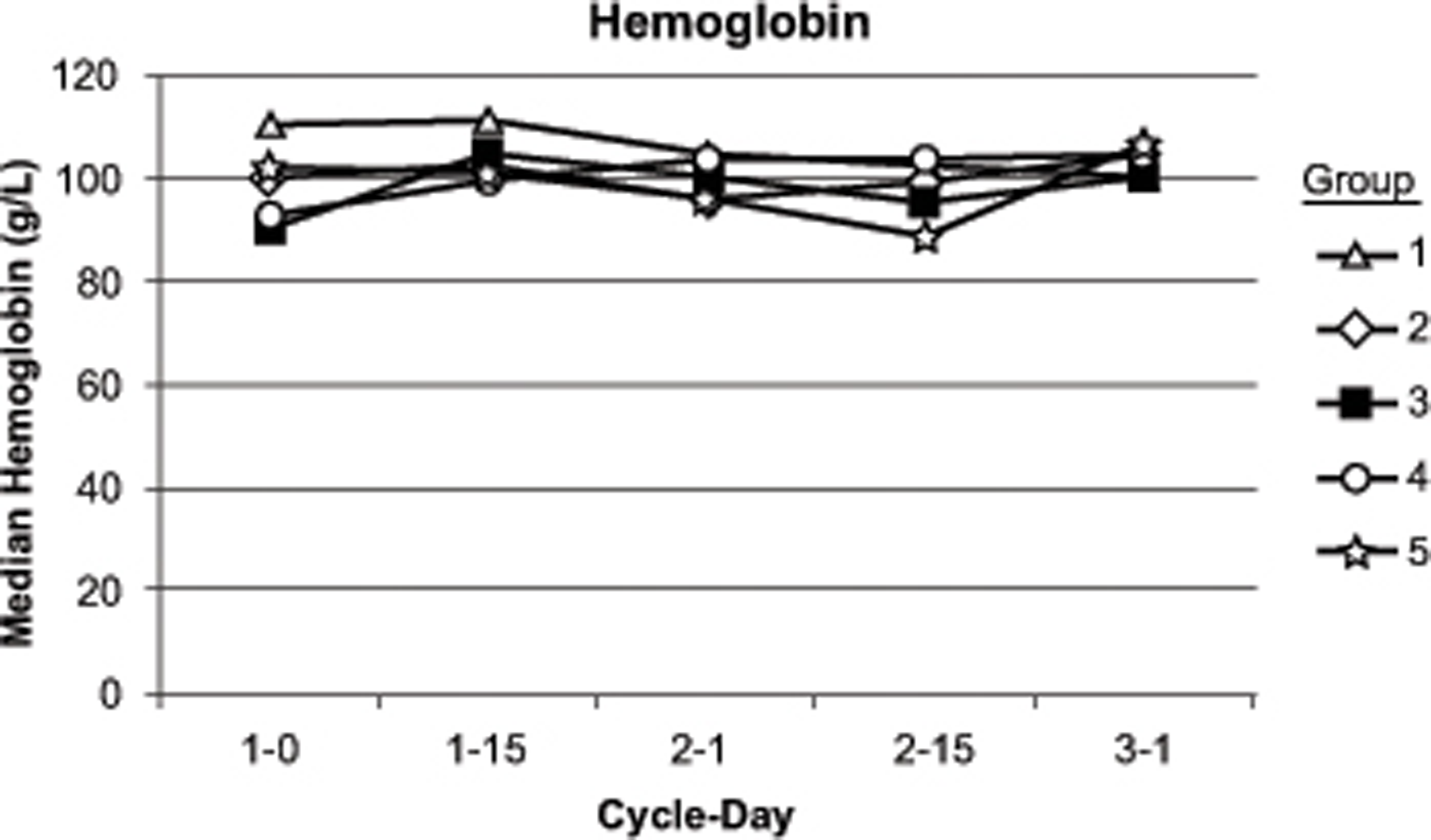

Supplement: Supplementary Figure 1D [file leu201329x4.tif]
