# Supplementary material for: Carfilzomib in multiple myeloma patients with renal impairment: pharmacokinetics and safety
Source: Leukemia. 2013 Mar 1;27(8):1707–14. doi: 10.1038/leu.2013.29 (PMC3740399; doi:10.1038/leu.2013.29)
Supplement: Supplementary Figure Legend [file leu201329x5.pdf]

Supplemental Figure 1. Hematological laboratory assessments during treatment with carfilzomib.

Neutrophil counts(A), platelet counts (B), white blood cell counts (C), and hemoglobin levels (D) were assessed by study site laboratories prior to dosing on Days 1 and 15 of Cycles 1 and 2, and Day 1 of Cycle 3. Points represent median values. Group 1, normal renal function; Group 2, mild renal impairment; Group 3, moderate renal impairment; Group 4, severe renal impairment; Group 5, chronic dialysis.
