# Supplementary material for: Carfilzomib in multiple myeloma patients with renal impairment: pharmacokinetics and safety
Source: Leukemia. 2013 Mar 1;27(8):1707–14. doi: 10.1038/leu.2013.29 (PMC3740399; doi:10.1038/leu.2013.29)
Supplement: Supplementary Table 1 [file leu201329x6.pdf]

Supplemental Table 1. Extent of Carfilzomib Exposure

	**Group 1 (n=12)**	**Group 2 (n=12)**	**Group 3 (n=10)**	**Group 4 (n=8)**	**Group 5 (n=8)**	**Total (N=50)**
Treatment Duration (days), median (range)	148.5 (38–325)	134.5 (36–310)	58 (14–337)	274.5 (16–332)	65 (9–247)	96 (9–337)
Last Cycle Started, median (range)	5.5 (2–12)	5.5 (2–11)	2.5 (1–12)	10 (1–12)	3 (1–9)	4 (1–12)
Cumulative Dose Across Cycles (mg/m^2), median (range)	664.5 (130–1704)	637.5 (150–1218)	281 (90–1270)	959.5 (90–1518)	280 (60–1351)	416 (60–1704)

Supplemental Figure 1. Hematological laboratory assessments during treatment with carfilzomib.

Neutrophil counts(A), platelet counts (B), white blood cell counts (C), and hemoglobin levels (D) were assessed by study site laboratories prior to dosing on Days 1 and 15 of Cycles 1 and 2, and Day 1 of Cycle 3. Points represent median values. Group 1, normal renal function; Group 2, mild renal impairment; Group 3, moderate renal impairment; Group 4, severe renal impairment; Group 5, chronic dialysis.

A

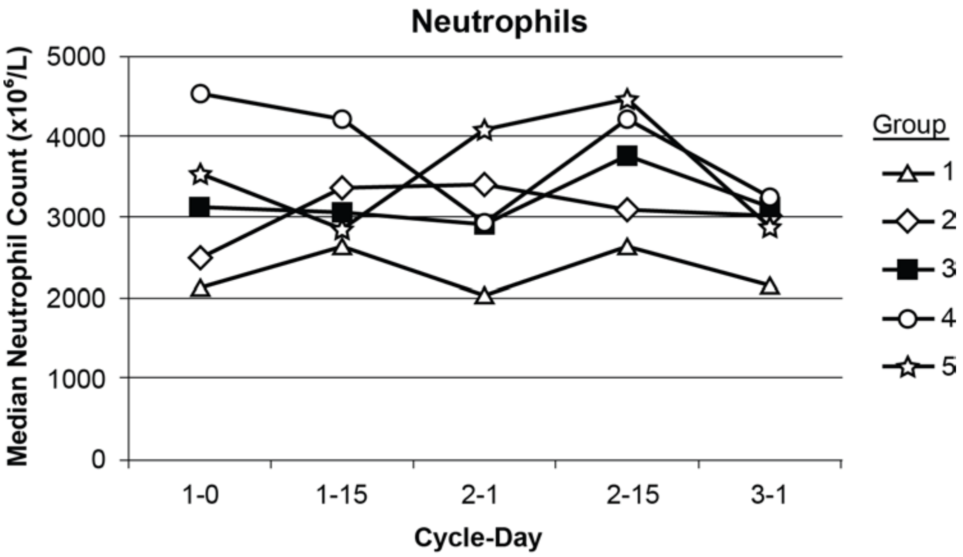
Neutrophils
Median Neutrophil Count (x10⁶/L)
5000
4000
3000
2000
1000
0
1-0
1-15
2-1
2-15
3-1
Cycle-Day
Group
1
2
3
4
5

B

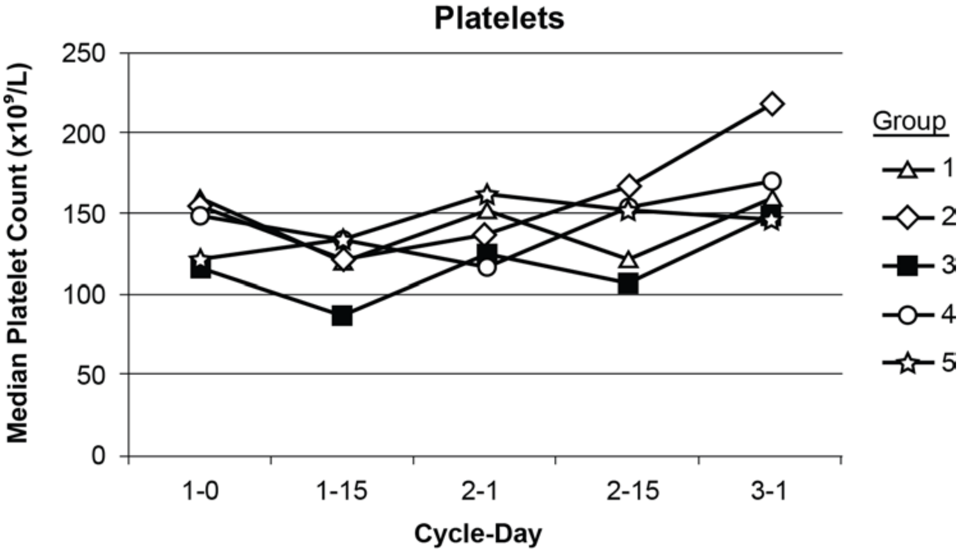
Platelets
Median Platelet Count (x10⁹/L)
250
200
150
100
50
0
1-0
1-15
2-1
2-15
3-1
Cycle-Day
Group
1
2
3
4
5

C

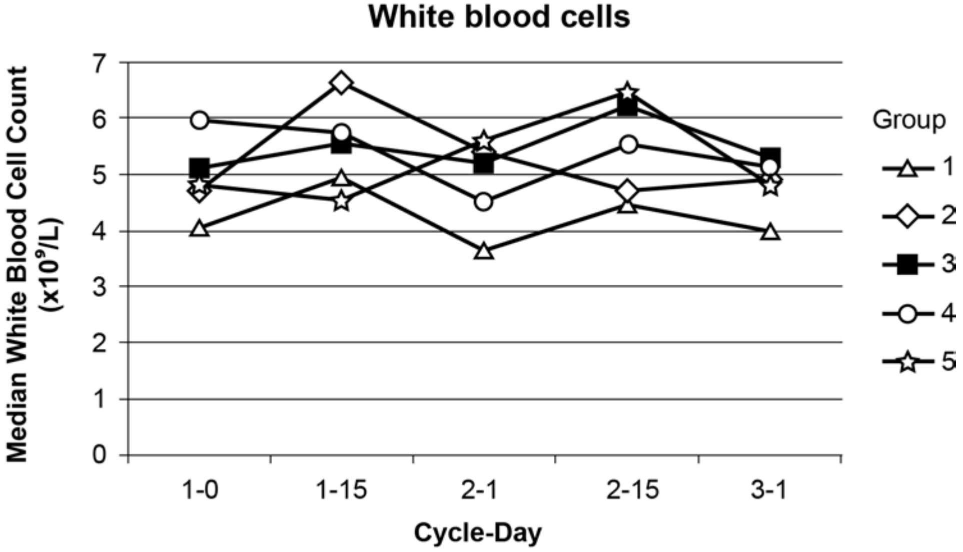
White blood cells
Median White Blood Cell Count ($x10^9$/L)
7
6
5
4
3
2
1
0
1-0
1-15
2-1
2-15
3-1
Cycle-Day
Group
1
2
3
4
5

D

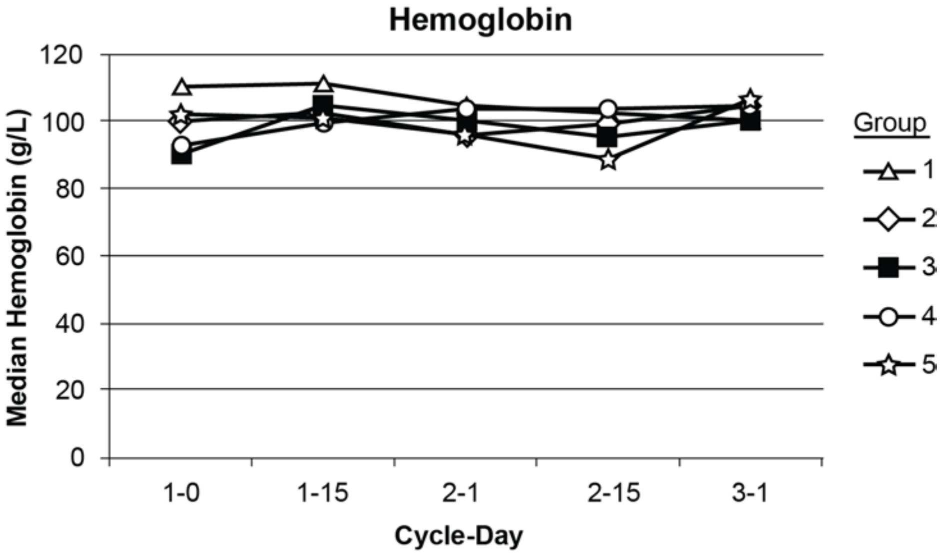
Hemoglobin
Median Hemoglobin (g/L)
120
100
80
60
40
20
0
1-0
1-15
2-1
2-15
3-1
Cycle-Day
Group
1
2
3
4
5
